# Suicide attempts in clinical trials with paroxetine randomised against placebo

**DOI:** 10.1186/1741-7015-3-14

**Published:** 2005-08-22

**Authors:** Ivar Aursnes, Ingunn Fride Tvete, Jorund Gaasemyr, Bent Natvig

**Affiliations:** 1 Department of Pharmacotherapeutics, University of Oslo, Oslo, Norway; 2 Department of Mathematics, University of Oslo, Oslo, Norway

## Abstract

**Background:**

Inclusion of unpublished data on the effects of antidepressants on children has suggested unfavourable risk-benefit profiles for some of the drugs. Recent meta-analyses of studies on adults have indicated similar effects. We obtained unpublished data for paroxetine that have so far not been included in these analyses.

**Methods:**

The documentation for drug registration contained 16 studies in which paroxetine had been randomised against placebo. We registered the number of suicides, suicide attempts and ideation. We corrected for duration of medication and placebo treatment and used a standard Bayesian statistical approach with varying priors.

**Results:**

There were 7 suicide attempts in patients on the drug and 1 in a patient on placebo. We found that the probability of increased intensity of suicide attempts per year in adults taking paroxetine was 0.90 with a "pessimistic" prior, and somewhat less with two more neutral priors.

**Conclusion:**

Our findings support the results of recent meta-analyses. Patients and doctors should be warned that the increased suicidal activity observed in children and adolescents taking certain antidepressant drugs may also be present in adults.

## Background

The debate about whether the use of antidepressant drugs increases suicidal activity has recently been sharpened after more than 10 years of turmoil [1]. Conclusions concerning children and adolescents have been drawn [2]. Inclusion of unpublished data suggested unfavourable risk-benefit profiles for some of the drugs. For adults, industry points to the absence of data refuting the null hypothesis (no such increase).

In a February 19^th ^BMJ editorial [3] accompanying two meta-analyses of suicidal activities in adult patients on SSRIs [4,5], the authors failed to convey the unanimous conclusion in the reviewed studies of an increased risk of suicidal attempts. Admittedly, one of the analyses only touched statistical significance, but that might have been due to the withholding of data by the manufacturer of one of the drugs. We have had access to some of those missing data. Recently we were given the opportunity to review the clinical data on paroxetine as presented to the world's drug regulatory agencies in 1989. One of the published meta-analyses [4] contained summaries of the documentation provided by the marketing authorization holders to the MHRA, which did not distinguish between suicidal attempt and suicidal ideation for paroxetine. We studied the primary data and even the individual case descriptions when available. Another meta-analysis [5] reported published data only, whereas most of our data were from unpublished studies. Moreover, as opposed to the BMJ authors, we have based our statistical analysis on comparing intensities of suicide attempts per year in drug and placebo groups, taking the exposure time of the patients properly into account. We now present our findings and estimate the degree of support for the idea of an increased intensity per year of suicide attempts in adults.

## Methods

We included only double blind, parallel design studies with patients (all adults) randomised to either paroxetine or placebo. Altogether 16 studies met these criteria (references 79 to 93 and 95 in the Expert Report), containing respectively 916 and 550 paroxetine and placebo treated patients. The study period was in most instances 6 weeks. One important exception was a study (reference 91) with a preponderance of paroxetine use over placebo and lasting for 17 weeks. Patients were excluded from the studies after a suicide-related event. Taking this censoring into account, paroxetine treatment made up 190.7 patient years altogether and placebo 73.3 patient years. Suicide-related events could be found in tables in the Expert Report, in the adverse reactions section in the individual study reports, and in the individual patient descriptions.

We let θ_p _be the intensity per year of a suicide attempt in the placebo group and θ_d _the intensity per year in the drug group, for a random patient in the 16 studies; correspondingly, X_p _and X_d _represent the total numbers of suicide attempts. We can have at most one suicide attempt for each patient. Taking this censoring into account, we denoted the corresponding patient years in the 16 studies combined by m_p _and m_d_. In addition, patients in both the placebo and drug groups are supposed to behave in a similar manner. It then follows that the likelihood of the experiment corresponds to X_p _and X_d _having Poisson distributions respectively with parameters (m_p_θ_p_) and (m_d _θ_d_). In addition, we assume that the two variables were conditionally independent given the parameters. The corresponding observed data are (x_p, _m_p_) and (x_d, _m_d_), and the prior information is denoted by (x^o^_p_, m^o^_p_) and (x^o^_d_, m^o^_d_).

The Bayesian approach is based on the construction of probability distributions for θ_p _and θ_d _. This does not mean that these parameters are to be interpreted as random variables, but our knowledge of the parameters is uncertain and we describe this uncertainty with the help of probability distributions. Probability distributions describing our initial uncertainty are called prior distributions (that is, before real data are collected). When the real data are taken into account, the prior distributions are updated by Bayes' formula to posterior distributions. An excellent introduction to Bayesian methods in medicine is given by Spiegelhalter et al. [6].

We assume that the prior distribution for θ_p _is gamma, with parameters x^o^_p _and m^o^_p_, while correspondingly θ_d _has the parameters x^o^_d _and m^o^_d _and is assumed to be independent of the prior distribution for θ_p_. Hence, standard Bayesian theory gives the posterior distribution of θ_p _as gamma, with parameters x^o^_p _+ x_p _and m^o^_p _+ m_p, _while θ_d _will have the parameters x^o^_d _+ x_d _and m^o^_d _+ m_d_. We performed simulations by making 80000 random draws of θ_d _and θ_p _from their independent gamma posterior distributions, computed the logarithms of the ratios θ_d_/θ_p, _and constructed diagrams by applying a standard density estimation technique to these logarithms. (The logarithm was introduced to avoid an unwelcome feature of the density estimation method.) Note that the logarithm of the ratio θ_d_/θ_p _is greater than zero whenever θ_d _is greater than θ_p_. Hence, we calculated the probabilities that medication with paroxetine is associated with an increased intensity of a suicide attempt per year as the proportions of logarithmic ratios greater than zero in the samples. This corresponds to areas below the densities to the right of zero in the diagrams.

The grounds for a pessimistic prior have been given by Healy and Whitaker [7] who, relating the occurrence of suicidal activities to the use of antidepressant drugs, estimated an odds ratio of 2.4 from evidence given in clinical trials, epidemiological observations and case histories. The clinical trial data they used included, but were not restricted to, studies with the active drugs randomised against placebo. Mathematically, we chose to express this view as equivalent to observing two (x^o^_d_) events with paroxetine during 50 (m^o^_d_) patient years and one (x^o^_p_) with placebo during 50 (m^o^_p_) patient years, adding up to 3 attempts per 100 patient years, which is similar to our observed average value for paroxetine and placebo taken together. We based the calculations on a total of only 100 (m^o^_d _+ m^o^_p_) patient years in the prior, compared to 264 (m_d _+ m_p_) patient years in the real data, in order to increase the importance of the real data over the prior information. The slightly optimistic and slightly pessimistic priors represent respectively a paper by Lapierre [8] (appearing in tandem with Healy and Whitaker) and the article that reported suicidal ideation in children medicated with paroxetine [2]. The former author took the attitude that, if anything, there were slight signs of reduced suicidal activity connected with antidepressants, whereas the latter authors left the reader with the assumption that the observed increased suicidal ideation in children must somehow be reflected in adults. We assigned the numbers of suicidal patients on paroxetine and placebo per 50 patient years to be respectively 1.35 and 1.65 and *vice versa*.

## Results

There were no suicides in the 16 studies with paroxetine randomised against placebo. Suicidal activities are listed in table 1. Summarising the suicide attempts, there are seven among the patients on paroxetine and one among the patients on placebo. (One event tabulated in the Expert Report as occurring with placebo did in fact happen during the run-in period before randomisation.)

**Table 1:** Suicide attempts and ideation in randomised clinical trials with paroxetine against placebo. Extracted from "APPLICATION FOR MARKETING AUTHORIZATION: SEROXAT" 1989. * Referring to list in Part I, Volume 3

**Patient identification number**	Study reference number*	**Suicidal attempt**	**Suicidal ideation**	**Medication**	**Tabulated**	**Individually described**
02 01 009	79	X		Placebo	Yes	Yes
02 04 089	82	X		Paroxetine	Yes	Yes
03 002 034	84		X	Placebo	No	Yes
04 02 056	84	X		Paroxetine	Yes	Yes
1 09 021	90	X		Washout	Yes	Yes
09 01A 005	91	X		Paroxetine	Yes	Yes
09-01A-006	91		X	Paroxetine	No	Yes
09 01E 260	91	X		Paroxetine	Yes	No
09 01J 573	91	X		Paroxetine	Yes	Yes
09-OU-620	91		X	Paroxetine	No	Yes
09-01G-405	91		X	Paroxetine	No	Yes
037	93	X		Paroxetine	No	Yes
07 01A 001	95	X		Paroxetine	Yes	Yes

**Figure 1:**
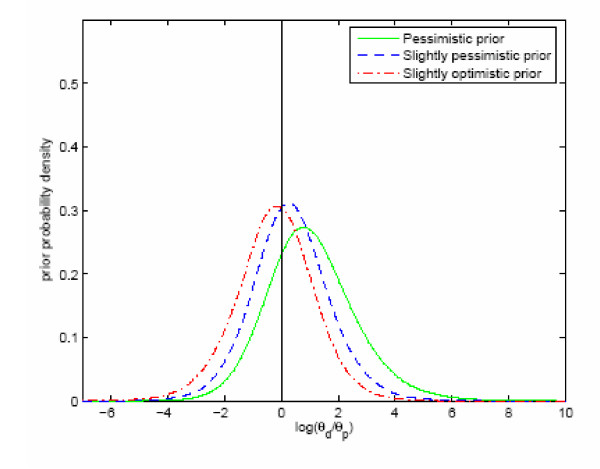
**Prior intensity of suicidal attempt. **Distributions of three different prior (see text) logarithmic intensity ratios ln(θd/θp) (logarithmic intensity of a suicide attempt on drug minus logarithmic intensity of a suicide attempt on placebo).

**Figure 2:**
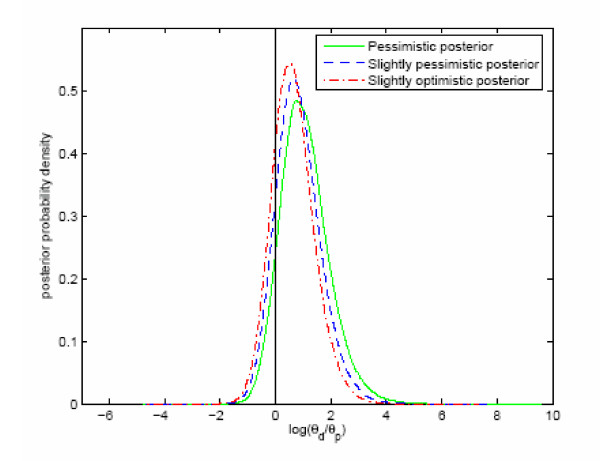
**Posterior intensity of suicidal attempt. **Distributions of posterior logarithmic intensity ratios ln(θ_d_/θ_p_) with three different priors.

The three prior distributions of the logarithm of the ratio θ_d_/θ_p _are shown in figure 1, and the corresponding posterior distributions are shown in figure 2. The probability that medication with paroxetine is associated with an increased intensity per year of a suicide attempt is 0.90 with the pessimistic prior (Healy and Whitaker [7]), and 0.79 (Lapierre [8]) and 0.85 (Whittington *et al. *[2]) with the two other priors. The corresponding prior probabilities were respectively 0.75, 0.42 and 0.58.

## Discussion

We believe that the chosen studies are similar enough to be pooled for analysis. This view is supported by the similarities of the protocols for the various studies, although the populations that were studied differed considerably. We also believe it is best to count patient years rather than patients, although claims have been made for the contrary [7]. At least, counting patient years is a more conservative approach. Furthermore, to treat the patient as a unit and to assume binomial distributions would be inappropriate since patients have different follow-up times and hence different probabilities of suicide attempts within both the placebo and the drug groups.

Another statistical approach would have been to express the prior distributions in terms of independent priors for θ_p _and the ratio θ_d_/θ_p_, with a common, relatively weak prior for θ_p _in all three formulations. This was our approach in a previous publication using basically the same method [9]. To make things simpler and perhaps more transparent in this short paper, we have not used this approach here.

## Conclusion

Although we report a small data set, by taking various priors into account the data strongly suggest that the use of SSRIs is connected with an increased intensity of suicide attempts per year. The two meta-analyses and our contribution taken together make a strong case for the conclusion, at least with a short time perspective, that adults taking antidepressants have an increased risk of suicide attempts. We also conclude that the recommendation of restrictions on the use of paroxetine for children and adolescents recently conveyed by regulatory agencies [10] should be extended to include usage by adults.

## Competing interests

The author(s) declare that they have no competing interests.

## Authors' contributions

IA collected the data, presented the problem and drafted the manuscript along with BN, who suggested the statistical solution based on earlier work by the present authors [9]; IFT did the computations and took part in the planning along with JG. All authors read and approved the final manuscript.

## Pre-publication history

The pre-publication history for this paper can be accessed here:


